# Assessment of neutrophil percentage to albumin ratio as a diagnostic biomarker for osteoarthritis: A cross-sectional study utilizing NHANES data

**DOI:** 10.1097/MD.0000000000045681

**Published:** 2025-11-07

**Authors:** Qiangqiang Lian, Yimin Liu, Faming Tian, Yunpeng Hu, Hetong Li, Hongmei Ding, Liu Zhang

**Affiliations:** a School of Public Health, Hebei Key Laboratory for Organ Fibrosis Research, North China University of Science and Technology, Tangshan, Hebei, China; b North China University of Science and Technology, Tangshan, Hebei, China; c Department of Orthopedic Surgery, Hebei Medical University, Shijiazhuang, China; d Department of Orthopaedics, Shenzhen Hospital, Southern Medical University, Shenzhen; e Department of Clinical Laboratory, Tianjin Hospital, Tianjin University, Tianjin, China; f Department of Orthopedic Surgery, Emergency General Hospital, Beijing, China.

**Keywords:** biomarker, diagnosis, neutrophil percentage to albumin ratio, osteoarthritis

## Abstract

Osteoarthritis (OA) is a major global public health issue that significantly impairs patients’ quality of life. The neutrophil percentage to albumin ratio (NPAR) has emerged as a promising inflammatory marker for the diagnosis and prognosis of various inflammatory conditions, yet its relationship with OA remains unexplored. This study aimed to examine the association between NPAR and OA and evaluate its diagnostic potential. Using cross-sectional data from the National Health and Nutrition Examination Survey (NHANES, 1999–2020), we analyzed 38,302 participants, including 2890 with OA. Weighted multivariate logistic regression, subgroup analyses, curve fitting, and inflection point analyses were employed, with C-reactive protein as a positive control. Diagnostic performance was assessed via receiver operating characteristic curve analysis. After adjusting for covariates, elevated NPAR was significantly associated with increased OA risk (odds ratio [OR] = 1.045; 95% confidence interval [CI]: 1.016–1.075; *P* = .0029). Subgroup analyses indicated significant associations in males (OR = 1.071; 95% CI: 1.024–1.121; *P* = .003), individuals aged 20 to 49 (OR = 1.057; 95% CI: 1.001–1.115; *P* = .045) and 60 to 69 (OR = 1.064; 95% CI: 1.003–1.130; *P* = .041), Mexican Americans (OR = 1.115; 95% CI: 1.016–1.223; *P* = .022), non-Hispanic whites (OR = 1.043; 95% CI: 1.009–1.079; *P* = .014), and moderate drinkers (OR = 1.117; 95% CI: 1.050–1.189; *P* < .001). Diagnostic evaluation revealed that NPAR had lower overall accuracy than C-reactive protein; however, among males, its diagnostic performance was comparable. In conclusion, NPAR is positively associated with OA risk and may offer supplementary diagnostic value, particularly in populations where C-reactive protein is less sensitive.

## 1. Introduction

Osteoarthritis (OA) is a chronic, progressive joint disorder marked by articular cartilage degeneration and bone hyperplasia at joint margins.^[1]^ OA significantly affects patients’ daily quality of life and represents a major global public health concern due to its high prevalence and associated chronic pain.^[2]^ The World Health Organization identifies OA as a major cause of disability, impacting over 10% of adults globally.^[3,4]^ The rising occurrence of OA driven by an aging population underscores the critical need for early diagnostic and treatment approaches.^[4–6]^

The diagnosis of OA typically relies on clinical assessment, imaging studies, and laboratory tests.^[2,7,8]^ However, due to the complexity and cost of these procedures, patients may be less willing to undergo testing, potentially leading to missed opportunities for early diagnosis and treatment. In recent years, the neutrophil percentage and albumin ratio (NPAR), as a cost-effective and easily accessible inflammatory biomarker, has shown potential value in the diagnosis and prognostic assessment of various diseases.^[9–11]^

NPAR is derived by calculating the ratio of the neutrophil percentage to albumin in the blood, serving as an indicator of systemic inflammatory status.^[12]^ In inflammatory diseases similar to OA, elevated NPAR levels have been associated with disease severity and inflammatory activity.^[12–14]^ The relationship between NPAR and OA remains unclear, and further research is needed to determine the diagnostic value of NPAR in OA.

This study aims to further explore the association between NPAR and the occurrence of OA and to assess its potential as a diagnostic biomarker. This study analyzes National Health and Nutrition Examination Survey (NHANES) data from 1999 to 2020 to offer new insights into NPAR’s role in OA, potentially aiding clinical diagnosis and treatment.

## 2. Methods

### 2.1. Data sources

**Figure 1.**
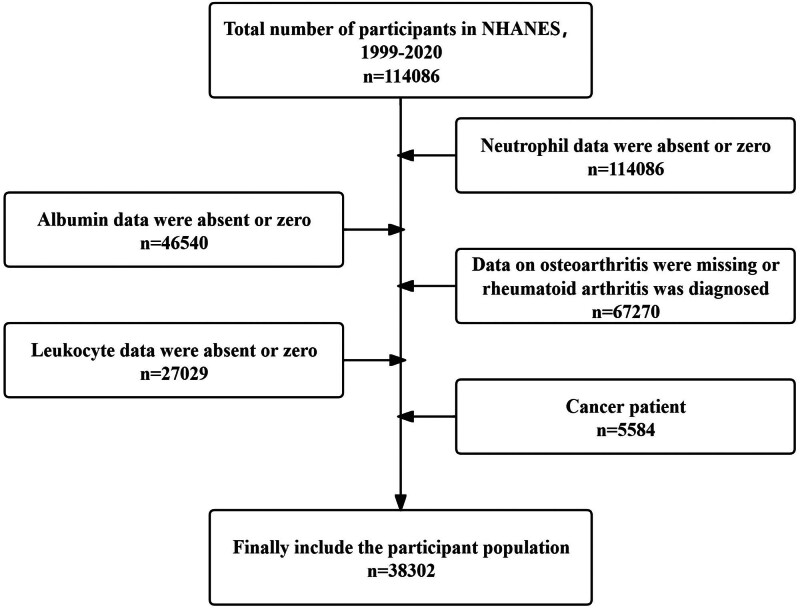
Study cohort selection flowchart.

This retrospective cross-sectional study utilizes data from the NHANES spanning 1999 to 2020. The NHANES, conducted by the National Center for Health Statistics under the Centers for Disease Control and Prevention, evaluates the health and nutritional status of the US noninstitutionalized population. The survey employs a multistage sampling design to ensure national representativeness of the data. After a household interview, participants receive thorough evaluations, encompassing physical exams, specialized assessments, and laboratory tests, at the Mobile Examination Center. The research data are publicly available, and researchers have obtained permission for use, ensuring the reliability and comprehensiveness of the assessment.^[15]^ Ethics review committee approval is available on the NHANES website (https://wwwn.cdc.gov/nchs/nhanes/default.aspx).The study included 38,302 subjects after excluding those with missing or zero values for neutrophil counts, albumin levels, white blood cell counts, OA-related data, and those diagnosed with cancer (Fig. 1).

### 2.2. Measurement of NPAR indicator

Hematological parameters were measured using the Beckman Coulter DxH 900 automated hematology analyzer following the NHANES CBC Profile. This device can measure red and white blood cell counts, hemoglobin, hematocrit, and red cell indices. White blood cell differentials were completed using the Coulter VCS system. The DxH 900 system features automatic dilution and mixing capabilities, as well as a single-beam photometer for hemoglobin measurement. NPAR was calculated as the ratio of the neutrophil percentage to the albumin concentration.^[16]^

### 2.3. Diagnosis of OA

OA diagnosis data were sourced from the NHANES “Medical Conditions” questionnaire. First, participants were asked if a doctor had ever told them that they had arthritis. If participants answered “yes,” they were prompted to specify the type of arthritis, such as OA, rheumatoid arthritis, or psoriatic arthritis, as classified by the NHANES questionnaire.^[17]^

### 2.4. Covariates

Demographic information such as age, gender, race, poverty-income ratio (PIR), and education level was collected through face-to-face interviews conducted by trained NHANES interviewers using the Household and Sample Demographic Questionnaire and the Computer-Assisted Personal Interviewing system. Participants were categorized as nonsmokers if they had a lifetime history of smoking fewer than 100 cigarettes and as smokers if they had smoked more than 100 cigarettes in their lifetime. Alcohol consumption was classified into 3 categories: excessive drinking (≥3 drinks for women, ≥4 drinks for men), moderate drinking (2 drinks for women, 3 drinks for men), and light drinking (≤1 drink for women, ≤2 drinks for men). Data on C-reactive protein (CRP), total cholesterol (defined as hyperlipidemia if ≥240 mg/dL), phosphorus, calcium, glucose, and creatinine were sourced from NHANES laboratory files. Body mass index (BMI) was derived from NHANES body measurement data for statistical analysis. Covariate selection was based on OA risk factors and NPAR confounders previously reported in the literature.^[18–20]^

All statistical analyses utilized SAS Survey Analysis Procedures to account for the NHANES database’s complex sampling design, ensuring nationally representative estimates. We utilized the weighted samples, strata, and clusters from the NHANES database and employed the SURVEY procedures in SAS to calculate the weighted population totals for this study. Continuous variables are reported as weighted means and standard errors, while categorical variables are reported as unweighted counts and weighted proportions. The samples were weighted following National Center for Health Statistics analytical guidelines.

A weighted multivariate logistic regression analysis was conducted to examine the relationship between NPAR and OA incidence. Subgroup analysis was subsequently conducted to further reveal possible population differences. The relationship between NPAR and OA occurrence was evaluated using smooth curve fitting and inflection point analysis. Additionally, this study compared the diagnostic efficacy of NPAR. The study incorporated 3 models: Model 1 was unadjusted, Model 2 was adjusted for age, gender, race, education level, marital status, and PIR, and Model 3 included adjustments for all covariates.

## 3. Results

### 3.1. Participant characteristics

This study included 38,302 participants, representing a weighted total of 154,481,783 US citizens. The participants had a mean age of 45.25 years, comprising 2890 individuals with OA and 34,412 without OA. Patients with OA had a significantly higher mean age (62.389 ± 13.826) compared with those without OA (43.854 ± 16.588). Demographic differences in gender, race, education level, and marital status were noted between the OA and non-OA groups, whereas the PIR showed no variation. The OA group exhibited significantly higher rates of mild alcohol consumption, smoking, obesity, and hyperlipidemia compared with the non-OA group, indicating notable differences in lifestyle habits and BMI between the 2 groups. Statistical differences were observed between the OA and non-OA groups for CRP, calcium, blood glucose, blood urea nitrogen, creatinine, albumin, and NPAR, whereas neutrophils, white blood cells, and phosphorus showed no significant differences (Table 1).

**Table 1:** Participants had baseline characteristics based on whether or not they had osteoarthritis.

Characteristic	Level	Overall	Normal	Osteoarthritis	*P*
		N = 154,481,783.08	N = 144,150,922.9	N = 10,330,860.18	
		n = 38,302	n = 35,412	n = 2890	
Gender	Man	19,176.00 (50.07)	18,064.00 (51.01)	1112.00 (38.48)	< .0001
	Female	19,126.00 (49.93)	17,348.00 (48.99)	1778.00 (61.52)	
Age		45.253 ± 17.111	43.854 ± 16.588	62.389 ± 13.826	< .0001
Race	Mexican American	7392.00 (19.30)	7053.00 (19.92)	339.00 (11.73)	< .0001
	Other Hispanic	3428.00 (8.95)	3221.00 (9.10)	207.00 (7.16)	
	Non-Hispanic White	15,393.00 (40.19)	13,771.00 (38.89)	1622.00 (56.12)	
	Non-Hispanic Black	8030.00 (20.96)	7452.00 (21.04)	578.00 (20.00)	
	Other race - including multiracial	4059.00 (10.60)	3915.00 (11.06)	144.00 (4.98)	
Education	Junior high school and below	9761.00 (25.51)	8973.00 (25.37)	788.00 (27.29)	.0001
	Senior high school	8750.00 (22.87)	8031.00 (22.71)	719.00 (24.90)	
	Above high school	19,747.00 (51.62)	18,367.00 (51.93)	1380.00 (47.80)	
Marital status	Married/living with a partner	20,000.00 (61.61)	18,593.00 (61.94)	1407.00 (57.66)	< .0001
	Widowed/divorced/separated	5846.00 (18.01)	4980.00 (16.59)	866.00 (35.49)	
	Never married	6614.00 (20.38)	6447.00 (21.48)	167.00 (6.84)	
PIR	<1.5	12,452.00 (35.67)	11,511.00 (35.68)	941.00 (35.55)	.6473
	1.5–3.5	11,397.00 (32.64)	10,513.00 (32.58)	884.00 (33.40)	
	>3.5	11,063.00 (31.69)	10,241.00 (31.74)	822.00 (31.05)	
Drunk	Light drinking	11,256.00 (46.19)	10,291.00 (45.16)	965.00 (61.04)	< .0001
	Moderate drinking	5500.00 (22.57)	5160.00 (22.65)	340.00 (21.51)	
	Overdrink	7611.00 (31.23)	7335.00 (32.19)	276.00 (17.46)	
Smoke	Yes	16,293.00 (42.57)	14,789.00 (41.79)	1504.00 (52.10)	< .0001
	No	21,980.00 (57.43)	20,597.00 (58.21)	1383.00 (47.90)	
BMI	Underweight to normal weight	11,789 (31.2)	11,206 (32.1)	583 (20.7)	< .001
	Overweight	13,067 (34.6)	12,092 (34.6)	975 (34.5)	
	Obesity	12,901 (34.2)	11,636 (33.3)	1265 (44.8)	
Hyperlipidemia	Yes	5157 (13.5)	4706 (13.3)	451 (15.6)	< .001
	No	33,124 (86.5)	30,685 (86.7)	2439 (84.4)	
C-reactive protein(mg/dL)		0.4 ± 0.8	0.4 ± 0.8	0.5 ± 0.9	< .001
Blood urea nitrogen(mg/dL)		13.09 ± 5.51	12.87 ± 5.30	15.76 ± 7.17	< .0001
phosphorus(mg/dL)		3.35 ± 1.20	3.35 ± 1.21	3.39 ± 1.19	.1363
Calcium(mg/dL)		9.39 ± 0.46	9.39 ± 0.46	9.43 ± 0.40	< .0001
Glucose(mg/dL)		99.38 ± 36.71	98.90 ± 36.67	105.29 ± 36.78	< .0001
Creatinine(mg/dL)		0.79 ± 0.49	0.79 ± 0.483	0.85 ± 0.55	< .0001
Neutrophil (1000 cell/uL)		4.29 ± 1.80	4.29 ± 1.81	4.34 ± 1.74	.1492
Leukocyte (1000 cell/uL)		7.25 ± 2.24	7.26 ± 2.26	7.23 ± 2.11	.6066
Albumin(g/dL)		4.24 ± 0.37	4.25 ± 0.373	4.15 ± 0.34	< .0001
NPAR		13.81 ± 2.86	13.77 ± 2.87	14.29 ± 2.74	< .001
Cycle year	1999–2000	3142.00 (8.20)	2921.00 (8.25)	221.00 (7.65)	< .0001
	2001–2002	3576.00 (9.34)	3314.00 (9.36)	262.00 (9.07)	
	2003–2004	3310.00 (8.64)	2976.00 (8.40)	334.00 (11.56)	
	2005–2006	3393.00 (8.86)	3103.00 (8.76)	290.00 (10.03)	
	2007–2008	3869.00 (10.10)	3479.00 (9.82)	390.00 (13.49)	
	2009–2010	4218.00 (11.01)	3846.00 (10.86)	372.00 (12.87)	
	2011–2012	3670.00 (9.58)	3491.00 (9.86)	179.00 (6.19)	
	2013–2014	3873.00 (10.11)	3668.00 (10.36)	205.00 (7.09)	
	2015–2016	3769.00 (9.84)	3555.00 (10.04)	214.00 (7.40)	
	2017–2020	5482.00 (14.31)	5059.00 (14.29)	423.00 (14.64)	

BMI = body mass index; N = number of weighted persons; n = number of unweighted persons; NPAR = neutrophil percentage to albumin ratio; PIR = ratio of family income to poverty.

### 3.2. Association between NPAR and OA

All demographic data were included in the weighted multivariate logistic regression analysis to examine the association between NPAR and OA (Table 2). NPAR, as a continuous variable, showed a statistically significant association in Model 3 (odds ratio [OR] = 1.045; 95% confidence interval [CI]: 1.016–1.075; *P* = .0029), suggesting a 4.5% increase in OA risk per unit increase in NPAR. For analysis, NPAR was transformed from a continuous variable into a categorical variable using quartiles. The fourth quartile of NPAR showed a 27.4% higher incidence of OA compared with the first quartile [OR = 1.274; 95% CI: 1.005–1.615; *P* = .0456]. After adjustment in Model 3, each unit increase in CRP, a common OA indicator, was associated with a 4% higher risk [OR = 1.04; 95% CI: 1.009–1.073; *P *=  .0125]. When compared with the first quartile, the last 3 quartiles of CRP did not show statistically significant differences, but there was a trend toward difference (Trend test = 0.0293) (Table 3).

**Table 2:** Weighted multivariate logistic regression of NPAR.

		Model 1			Model 2			Model 3	
	OR	CI	*P*-value	OR	CI	*P*-value	OR	CI	*P*-value
NPAR	1.082	1.063, 1.102	< .001	1.05	1.029, 1.071	< .001	1.045	1.016, 1.075	.0029
Q1	Reference								
Q2	1.13	0.984, 1.298	.084	1.052	0.882, 1.255	.5708	1.189	0.963, 1.469	.1072
Q3	1.353	1.179, 1.552	< .001	1.052	0.889, 1.246	.5502	1.058	0.846, 1.323	.6169
Q4	1.775	1.556, 2.025	< .001	1.374	1.183, 1.595	< .001	1.274	1.005, 1.615	.0456
Trend test			< .001			< .001			.0319

Model 1: Not adjusted.

Model 2: Age, sex, race, education, marital status, PIR.

Model 3: Age, sex, race, education, marital status, PIR, smoking, alcohol consumption, BMI, blood urea nitrogen, phosphorus, calcium, glucose, creatinine.

BMI = body mass index, CI = confidence interval, OR = odds ratio, PIR = ratio of family income to poverty.

**Table 3:** Weighted multivariate logistic regression of C-reactive protein.

		Model 1			Model 2			Model 3	
	OR	CI	*P*-value	OR	CI	*P*-value	OR	CI	*P*-value
C-reactive protein	1.191	1.139, 1.246	< .001	1.179	1.112, 1.249	< .001	1.04	1.009, 1.073	.0125
Q1	Reference								
Q2	1.658	1.385, 1.985	< .001	1.333	1.096, 1.621	.0045	0.956	0.760, 1.202	.6971
Q3	1.959	1.662, 2.309	< .001	1.471	1.236, 1.750	< .001	1.058	0.848, 1.320	.6135
Q4	2.511	2.102, 2.998	< .001	1.814	1.463, 2.248	< .001	1.227	0.994, 1.516	.0566
Trend test			< .001			< .001			.0293

Model 1: Not adjusted.

Model 2: Age, sex, race, education, marital status, PIR.

Model 3: Age, sex, race, education, marital status, PIR, smoking, alcohol consumption, BMI, blood urea nitrogen, phosphorus, calcium, glucose, creatinine.

BMI = body mass index, CI = confidence interval, OR = odds ratio, PIR = ratio of family income to poverty.

### 3.3. Subgroup analysis

We conducted subgroup analyses to examine whether the relationship between NPAR and prevalent OA was modified by sex, age, race/ethnicity, BMI, alcohol consumption, or smoking status. The Benjamini–Hochberg false discovery rate procedure was used (*q* < 0.05 for significance; *P* < .05 and .05 ≤ q < 0.10 for marginal significance).

Subgroup analyses adjusted for false discovery rate revealed a distinct population-specific pattern and complementarity in the effects of NPAR and CRP on OA. The association of NPAR was almost exclusively concentrated in males: OR = 1.071 (*q* = 0.006) in this group, with no significant difference observed in females. Regarding age, associations were marginally significant in both the 20 to 49 and 60 to 69 year age groups (*q* = 0.075), suggesting that young adults and those in early older age may be more susceptible to NPAR fluctuations. By race/ethnicity, associations were within the marginally significant range for Mexican Americans and non-Hispanic whites (*q* = 0.055 and 0.070, respectively), while no clear trends were observed in other ethnicities. For alcohol consumption, only moderate drinkers demonstrated a robust association (*q* = 0.003), and this signal was independent of BMI, no significant associations were found across any BMI strata (Table 4). In contrast, significant associations for CRP were concentrated in females, non-Hispanic Whites, individuals with normal/underweight BMI, and current smokers (*q* ≤ 0.026), suggesting that it may more likely reflect the systemic inflammatory burden in females and low BMI groups. Moderate drinkers also showed significance (*q* = 0.012), while the 50 to 69 year age group was only marginally suggestive. Overall, NPAR and CRP exhibited complementarity across different sex and BMI contexts: NPAR was sensitive in males and insensitive to BMI, whereas CRP was sensitive in females and normal-weight individuals. Altogether, they provide complementary inflammatory insights for OA risk prediction (Table 5).

**Table 4:** Subgroup analysis.

Group	OR (95% CI)	*P*-value	FDR	*P*_interaction_
Gender				.461
Men	1.071 (1.024–1.121)	.003	0.006	
Female	1.037 (0.997–1.079)	.068	0.068	
Age				.635
20–49	1.057 (1.001–1.115)	.045	0.075	
50–59	1.024 (0.954–1.100)	.507	0.507	
60–69	1.064 (1.003–1.130)	.041	0.075	
70–79	1.023 (0.942–1.111)	.583	0.583	
>80	1.057 (0.966–1.157)	.221	0.295	
Race				.388
Mexican American	1.115 (1.016–1.223)	.022	0.055	
Other Hispanic	1.044 (0.928–1.174)	.471	0.471	
Non-Hispanic White	1.043 (1.009–1.079)	.014	0.07	
Non-Hispanic Black	1.049 (0.994–1.107)	.079	0.132	
Other race - including multiracial	0.983 (0.753–1.283)	.897	0.897	
BMI				.384
Underweight to normal weight	1.057 (0.986–1.134)	.116	0.134	
Overweight	1.015 (0.957–1.077)	.618	0.618	
Obesity	1.036 (0.994–1.080)	.089	0.134	
Drunk				.103
Light drinking	1.028 (0.993–1.064)	.117	0.176	
Moderate drinking	1.117 (1.050–1.189)	<.001	0.003	
Overdrink	1.015 (0.940–1.097)	.696	0.696	
Smoke				.536
Yes	1.044 (1.001–1.090)	.045	0.054	
No	1.049 (1.005–1.094)	.027	0.054	

CI = confidence interval, FDR = false discovery rate, OR = odds ratio.

**Table 5:** Subgroup analysis of C-reactive protein.

Group	OR (95% CI)	*P*-value	FDR	*P*_interaction_
Gender				.382
Men	1.1 (0.97~1.24)	.135	0.135	
Female	1.17 (1.03~1.32)	.014	0.028	
Age				.785
20–49	1.112 (0.969–1.276)	.129	0.161	
50–59	1.225 (0.979–1.532)	.075	0.135	
60–69	1.322 (0.996–1.755)	.054	0.135	
70–79	1.138 (0.887–1.460)	.304	0.38	
>80	0.890 (0.575–1.376)	.584	0.584	
Race				.512
Mexican American	0.913 (0.679–1.227)	.538	0.538	
Other Hispanic	1.121 (0.917–1.371)	.255	0.319	
Non-Hispanic White	1.182 (1.058–1.321)	.004	0.02	
Non-Hispanic Black	1.097 (0.859–1.399)	.451	0.451	
Other race - including multiracial	2.006 (0.900–4.469)	.086	0.215	
BMI				.05
Underweight to normal weight	1.273 (1.093–1.483)	.002	0.006	
Overweight	0.988 (0.817–1.194)	.896	0.896	
Obesity	1.176 (0.969–1.427)	.099	0.149	
Drunk				.101
Light drinking	1.147 (1.001–1.316)	.05	0.075	
Moderate drinking	1.431 (1.124–1.822)	.004	0.012	
Overdrink	0.924 (0.690–1.238)	.594	0.594	
Smoke				.598
Yes	1.149 (1.031–1.281)	.013	0.026	
No	1.172 (0.988–1.389)	.067	0.134	

CI = confidence interval, FDR = false discovery rate, OR = odds ratio.

### 3.4. Curve fitting and inflection point analysis of NPAR and OA prevalence

**Figure 2.**
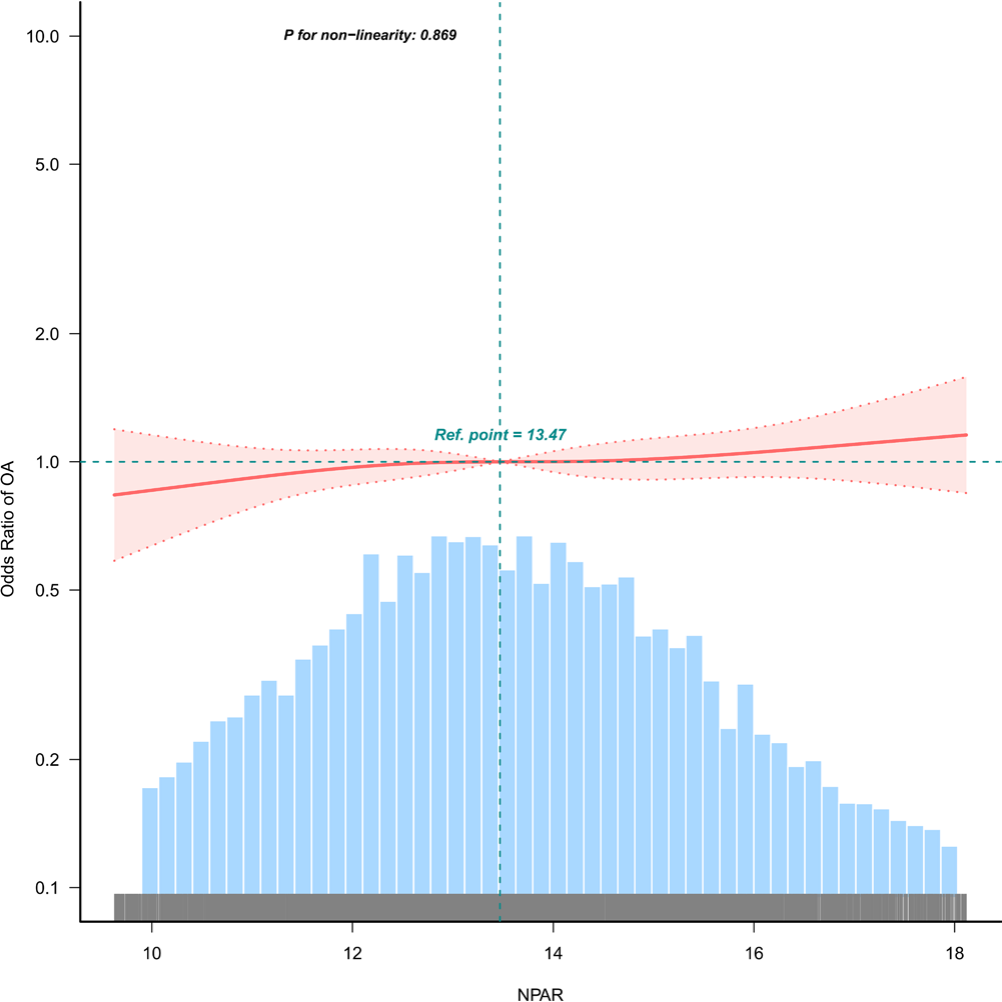
Curve fitting.

In this study, we performed curve fitting to evaluate the relationship between the variable and the primary outcome, focusing on NPAR and OA prevalence and identifying inflection points. The *P*-value for nonlinearity was .869, indicating a linear relationship between NPAR and the risk of OA (Fig.2). Inflection point analysis indicated no significant division in the linear relationship between NPAR and OA risk into distinct segments (Likelihood Ratio test = 0.759) (Table 6).

**Table 6:** Inflection point analysis.

Item	Breakpoint.OR (95% CI)	*P*-value
E_BK1	16.674 (16.276–17.072)	
slope1	1.017 (0.977–1.059)	.4064
slope2	1.034 (0.68–1.571)	.8757
Likelihood ratio test		.759
Nonlinear test		.869

CI = confidence interval, OR = odds ratio.

### 3.5. Diagnostic receiver operating characteristic curve analysis

The subgroup analysis compared receiver operating characteristic (ROC) results for NPAR and CRP across non-gender-specific, male, and female groups (Table 7).The study found that the area under the curve (AUC) for CRP (0.581) was significantly larger than that for NPAR (0.553) without gender differentiation (*P* < .0001). In males, the AUC for NPAR (0.607) did not significantly differ from that of CRP (0.586) (*P* = .066). In females, the AUC for CRP (0.559) significantly exceeded that for NPAR (0.504) (*P* < .0001).

**Table 7:** Diagnostic ROC analysis.

Gender	Variable	AUC	95 CI%	Youden Index	Sensitivity	Specificity	*P*-value
All	NPAR	0.553	(0.541–0.564)	0.087	0.645	0.442	<.0001
	C-reactive protein	0.581	(0.570–0.592)	0.120	0.677	0.442	
Men	NPAR	0.607	(0.589–0.626)	0.174	0.618	0.556	.066
	C-reactive protein	0.586	(0.568–0.604)	0.129	0.622	0.507	
Female	NPAR	0.504	(0.489–0.519)	0.031	0.969	0.063	<.0001
	C-reactive protein	0.559	(0.544–0.574)	0.092	0.734	0.358	

Model 3: Age, sex, race, education, marital status, PIR, smoking, alcohol consumption, BMI, blood urea nitrogen, phosphorus, calcium, glucose, creatinine.

AUC = area under the curve, BMI = body mass index, CI = confidence interval, NPAR = neutrophil percentage to albumin ratio, PIR = ratio of family income to poverty, ROC = receiver operating characteristic.

## 4. Discussion

This cross-sectional study utilizes the NHANES database to investigate the relationship between the NPAR and OA, evaluating NPAR’s potential as a diagnostic biomarker for OA. The results showed a positive correlation between NPAR and the occurrence of OA, and this positive association remained statistically significant after adjusting for covariates. Furthermore, the study found that the positive association between NPAR and OA prevalence was linear, without an inflection point dividing it into 2 meaningful segments. By evaluating the diagnostic effectiveness of NPAR through ROC analysis, it was found that its overall diagnostic performance was weaker than that of CRP, which is a typical diagnostic indicator for OA. However, in males, the diagnostic effectiveness of NPAR for OA was comparable to that of CRP. This study offers novel insights into NPAR’s role in OA, potentially serving as a valuable reference for clinical diagnosis and treatment.

Although OA has traditionally been viewed as a degenerative joint disease, recent research has clearly established that low-grade systemic inflammation plays a key role in its pathogenesis.^[21,22]^ NPAR may serve as an indirect indicator of OA-related synovial inflammation, cartilage degradation, and osteophyte formation processes. This potential arises from its integration of 2 key pathways: neutrophil-mediated innate immune activation (e.g., pro-inflammatory factor release via neutrophil extracellular traps), and the chronic inflammatory state reflected by hypoalbuminemia (e.g., interleukin-6 and tumor necrosis factor-α suppressing albumin synthesis).^[23,24]^ Furthermore, obesity and metabolic syndrome (which are associated with elevated NPAR) exacerbate the inflammatory milieu in OA through adipokine secretion, including including interleukin-1β, from adipose tissue.^[25,26]^ Therefore, an elevated NPAR may signify increased systemic inflammatory burden rather than merely localized degenerative changes in individuals with OA.

NPAR, as a biomarker of inflammation and nutritional status, reflects an individual’s inflammatory activity and nutritional condition by assessing the proportion of neutrophils in the blood and serum albumin levels.^[9,27]^ Neutrophils are essential in inflammatory responses, with their elevation typically signifying inflammation.^[28–32]^ Albumin levels reflect nutritional status, and their decrease may be associated with malnutrition or chronic diseases.^[33–35]^ An elevation in NPAR can be caused by various factors, but its increase collectively suggests the occurrence of inflammatory responses and malnutrition.^[35,36]^

OA is a chronic condition marked by articular cartilage degeneration, where inflammation is pivotal to its development. It is now known that inflammation is involved in multiple aspects such as cartilage damage, synovial inflammation, bone remodeling, and inflammation of surrounding tissues.^[25]^ Inflammatory responses contribute to cartilage destruction, with cytokines such as tumor necrosis factor-α and including interleukin-1β intensifying cartilage degeneration and associated pain.^[22,37–39]^ Increased systemic inflammatory markers, such as CRP, indicate that OA may affect various bodily systems.^[40]^ Weighted multivariate logistic regression indicated that higher NPAR levels were linked to an increased risk of OA occurrence (OR = 1.045, 95% CI: 1.016–1.075, *P* = .0029). The quartile categorical variable for NPAR also demonstrated that an increase in NPAR is statistically associated with prevalent OA (OR = 1.274; 95% CI: 1.005–1.615; *P* = .0456).

In this study, the comparison between NPAR and CRP aimed to evaluate the potential application value of NPAR in diagnosing OA. Subgroup analysis revealed varying associations between CRP, NPAR, and OA across different populations. CRP exhibited specificity in the female population, but not in the male population; conversely, NPAR showed specificity in the male population. In terms of age, NPAR was statistically significant in the 20 to 49 and 60 to 69 age groups, while CRP did not show specificity in any age group. Regarding race, NPAR was statistically significant in Mexican Americans and non-Hispanic whites, whereas CRP was only statistically significant in non-Hispanic whites. In the subgroup analysis based on smoking status, CRP exhibited specificity in smokers but not in nonsmokers; NPAR was significant in both nonsmokers and smokers. These findings suggest that NPAR may provide a supplementary diagnostic tool for OA in cases where CRP is not sensitive to certain populations.

The study utilized a smooth curve fitting method to reveal a linear relationship between NPAR and OA prevalence. The inflection point analysis showed that the relationship between NPAR and OA was a positive linear relationship without a meaningful inflection point, indicating that continuous close attention should be paid to NPAR. This discovery provides clinicians with a new perspective, namely that different levels of NPAR and their potential impact on disease progression should be considered when assessing OA risk.

The ROC analysis results showed that, in the general population, when used as diagnostic biomarkers for OA, CRP had better diagnostic efficacy than NPAR. However, in the male population, there was no statistical difference in diagnostic efficacy between NPAR and CRP. Therefore, the clinical value of NPAR lies in its potential to supplement the diagnosis of OA in cases where CRP is not sensitive to certain populations.

The main strengths of this study lie in its use of the large-scale, nationally representative NHANES dataset, which enhances the generality and extrapolability of the results. The study utilized weighted multivariate logistic regression analysis, accounting for various potential confounding factors to enhance result reliability. Curve fitting and inflection point analysis enhanced the comprehension of the NPAR and OA prevalence relationship. The ROC analysis compared the diagnostic effects of NPAR and CRP in OA, demonstrating that NPAR has certain application value in diagnosing OA.

Despite these strengths, the study also has some limitations. This study has limitations inherent to its cross-sectional design, precluding the establishment of causality between NPAR and OA onset or progression. Future prospective cohort studies or Mendelian randomization analyses are warranted to validate its potential as a predictor of OA progression.^[41,42]^ Second, although we considered various potential confounding factors, there may still be unmeasured or inadequately controlled confounding factors. Furthermore, there may be some errors in the measurement of NPAR, although standardized measurement methods were used to minimize this error. This study primarily examined the adult population in the United States, limiting the applicability of its findings to other racial or regional groups.

## Author contributions

**Formal analysis:** Yimin Liu.

**Project administration:** Hongmei Ding, Liu Zhang.

**Resources:** Faming Tian.

**Software:** Yunpeng Hu, Hetong Li.

**Writing – original draft:** Qiangqiang Lian.
